# Complex Femoral Reconstruction in an Osteoporotic Elderly Patient: From Trochanteric Femoral Nailing Fixation to Hemiarthroplasty

**DOI:** 10.7759/cureus.89904

**Published:** 2025-08-12

**Authors:** Sujith Shahul, Bisher Tulimat, Humam Ali, Ali H Ismaeil, Habib Alismaily

**Affiliations:** 1 Orthopedics, Rashid Hospital, Dubai, ARE; 2 Medicine, Royal College of Surgeons in Ireland, Medical University of Bahrain, Manama, BHR; 3 Medicine, Ajman University, Ajman, ARE

**Keywords:** elderly trauma, geriatric trauma management, hip hemi-arthroplasty, inter-trochanteric fracture, malunion femur fracture, osteoporotic intertrochanteric fracture, peri-implant fracture, proximal femur fracture, revision hip surgery, trochanteric femoral nail

## Abstract

Hip fractures in elderly patients with poor bone quality carry a high risk of complications such as malunion and per-implant fractures, especially in the presence of pre-existing implants. We report an 82-year-old male patient with multiple comorbidities and prior distal femoral fixation in 2011 who initially underwent trochanteric femoral nailing (TFNA) for a right intertrochanteric fracture, resulting in good recovery. One year later, he sustained a new fall, resulting in a peri-implant fracture and a right distal radius fracture. CT imaging revealed an occult malunion of the prior femoral neck fracture, leading to mechanical instability. Given poor bone quality and overlapping hardware, he underwent hardware removal, plating, and conversion to uncemented hemiarthroplasty via a lateral approach. Postoperatively, he was mobilized with a walker but required a wheelchair for longer distances due to lower-limb weakness and limitations from the wrist injury. This case highlights the value of advanced imaging in detecting occult malunion and the need for individualized surgical planning in elderly patients with complex fractures and prior hardware. Conversion arthroplasty remains an effective solution for per-implant fractures and malunion, facilitating early mobilization and functional recovery in high-risk patients.

## Introduction

Hip fractures are a major cause of morbidity, mortality, and loss of independence in the elderly population, particularly in those with multiple comorbidities and compromised bone quality, such as osteoporosis or osteopenia [1,2]. Intertrochanteric fractures account for approximately 45% of all hip fractures and are typically managed with internal fixation devices such as the TFNA implant (Trochanteric Fixation Nail Advanced, DePuy Synthes, Warsaw, IN), which offers biomechanical advantages for osteoporotic bones by allowing intramedullary load-sharing and better control of fracture fragments [3,4]. Despite advances in implant design, complications such as malunion, nonunion, hardware failure, and per-implant fractures continue to pose significant challenges in this population [5-7].

Per-implant and periprosthetic fractures are particularly complex in elderly patients due to factors such as poor bone quality, pre-existing implants, and medical comorbidities, which complicate both diagnosis and surgical planning [8,9]. Importantly, malunion may remain occult on plain radiographs and only become evident through advanced imaging modalities such as computed tomography (CT), which are critical for assessing mechanical alignment and implant stability [10].

When per-implant fractures occur in the context of prior fixation failures or malunion, treatment often necessitates conversion to arthroplasty rather than further internal fixation to restore mechanical stability and facilitate early mobilization [11,12]. However, revision surgeries in these patients carry substantial technical and perioperative risks, highlighting the need for individualized, multidisciplinary management strategies [13].

This case report describes an elderly male patient with multiple comorbidities who sustained sequential femoral fractures complicated by malunion and per-implant fracture, ultimately necessitating hardware removal and conversion to hemiarthroplasty. The report emphasizes the diagnostic challenges, surgical decision-making, and management considerations in such complex scenarios.

## Case presentation

An 82-year-old male patient with a history of hypertension, diabetes, ischemic heart disease with prior coronary angioplasty, chronic kidney disease, COPD, and a previous internal fixation of the distal right femur in 2011, presented to the emergency department on 25 May 2024 after sustaining a low-energy fall while praying. He complained of right hip pain and was unable to bear weight. He denied loss of consciousness, dizziness, headache, or other symptoms. On examination, he was fully conscious and oriented with a Glasgow Coma Score of 15/15. There was mild swelling and tenderness over the right hip, with no open wounds. Neurovascular status was intact. 

**Figure 1:**
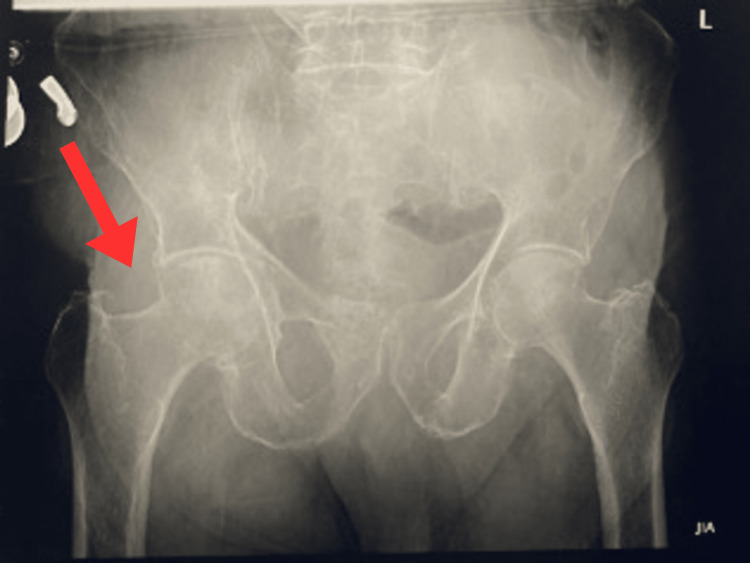
X-ray of the pelvis (AP view), 13 July 2018, showing normal proximal femoral anatomy prior to injury.

**Figure 2:**
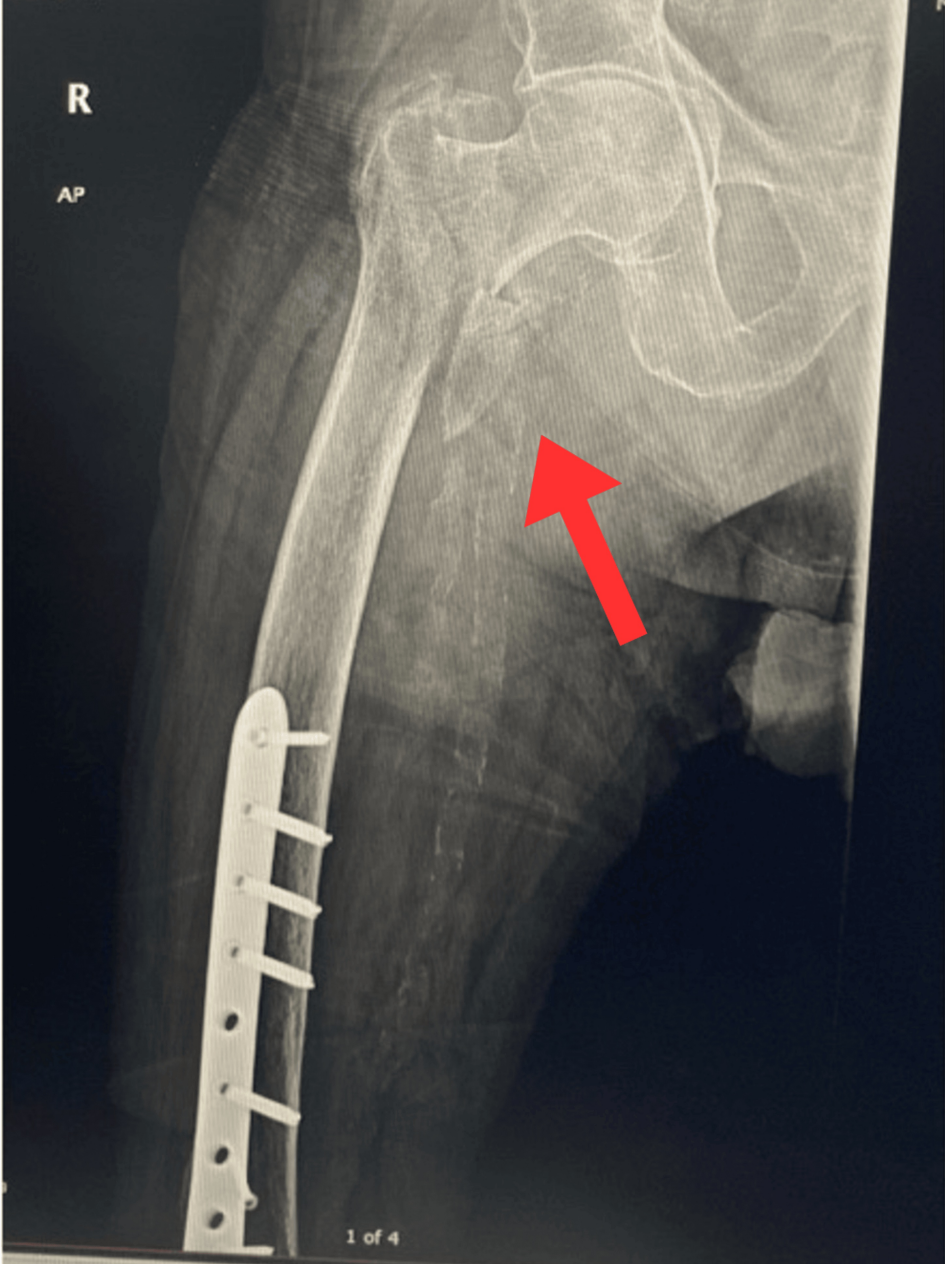
Preoperative X-ray of the right hip (AP view), 25 May 2024, demonstrating a comminuted displaced intertrochanteric fracture of the right proximal femur.

Figure 1 shows a prior radiograph that demonstrated an unremarkable right proximal femur with no fracture or hardware in the proximal segment. A later radiograph of the right hip, following the fall, revealed a comminuted, displaced intertrochanteric fracture of the right femur in the background of diffuse osteopenia, as shown in Figure 2. The patient was admitted under ortho-trauma for surgical planning. On 29 May 2024, he underwent closed reduction and internal fixation (CRIF) using a TFNA implant measuring 200 × 11 mm with a 130° angle, secured with a 95 mm lag screw and 35 mm distal locking screw. The fixation overlapped proximally with the previously locked distal femoral plate, and two proximal screws were replaced with new locking screws. Postoperatively, his neurovascular status was intact, and he was mobilized with physiotherapy.

**Figure 3:**
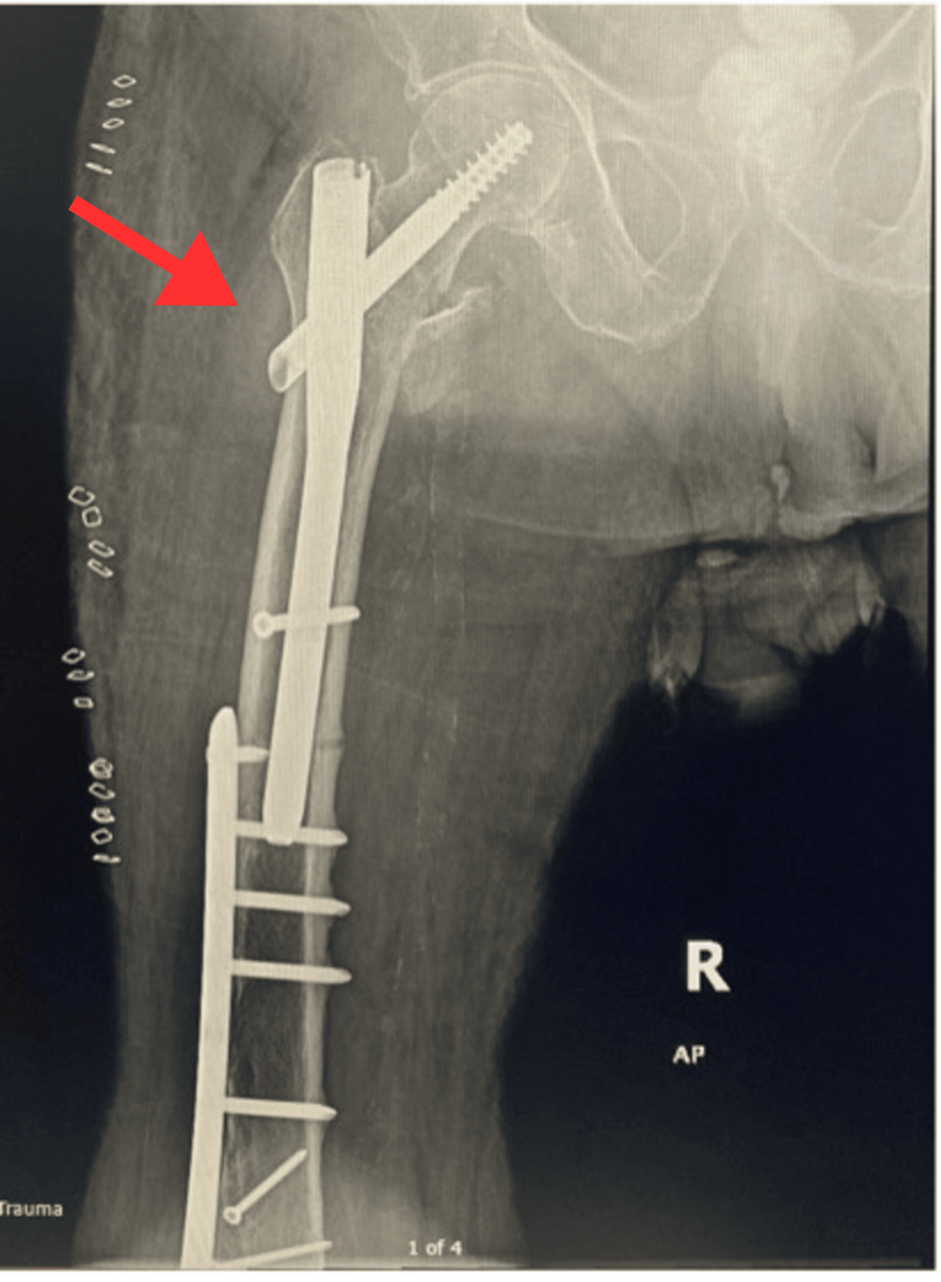
Postoperative right hip X-ray (AP view), 3 June 2024, showing TFNA fixation in place.

An X-ray was also taken showing satisfactory reduction and internal fixation of the intertrochanteric fracture (Figure 3). Overlap with the previously placed distal femoral plate is also noted. He was discharged on 6 June 2024 with instructions for gradual weight-bearing as tolerated and follow-up in the trauma clinic.

The patient was followed up in the clinic on 31 December 2024 and was reported to be doing well. The surgical wound was healed, with no signs of infection, and radiographs showed acceptable hardware positioning and fracture healing. 

**Figure 4:**
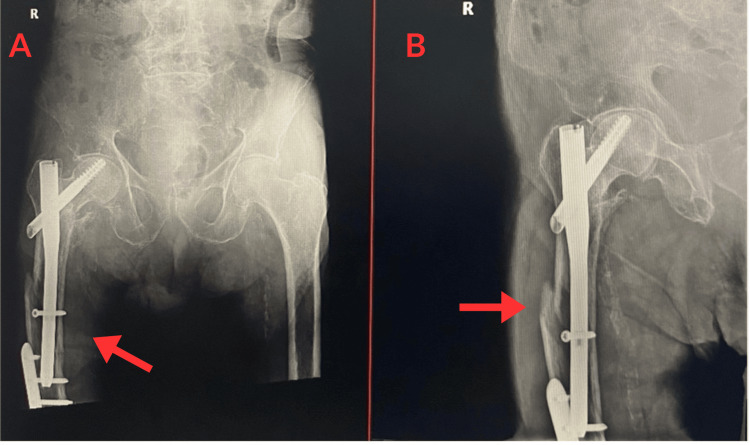
X-ray of the right hip (AP view), 17 July 2025, revealing an acute fracture in the proximal third of the right femur adjacent to the existing TFNA hardware. (A) and (B) demonstrate an acute fracture in the proximal third of the right femur (red arrows) adjacent to the TFNA hardware. Both images show the same fracture from slightly different exposures to enhance visualization of the fracture line.

Approximately one year later, in mid-June 2025, the patient presented again with new right hip pain following another fall. He reported feeling dizzy before the fall and sustained a right distal radius fracture, confirmed on radiographs, for which he was treated conservatively with a cast for six weeks. On arrival, he was alert and denied any loss of consciousness. An X-ray performed on 17 June 2025 showed an acute fracture in the proximal third right femur as shown in Figure 4A-4B.

**Figure 5:**
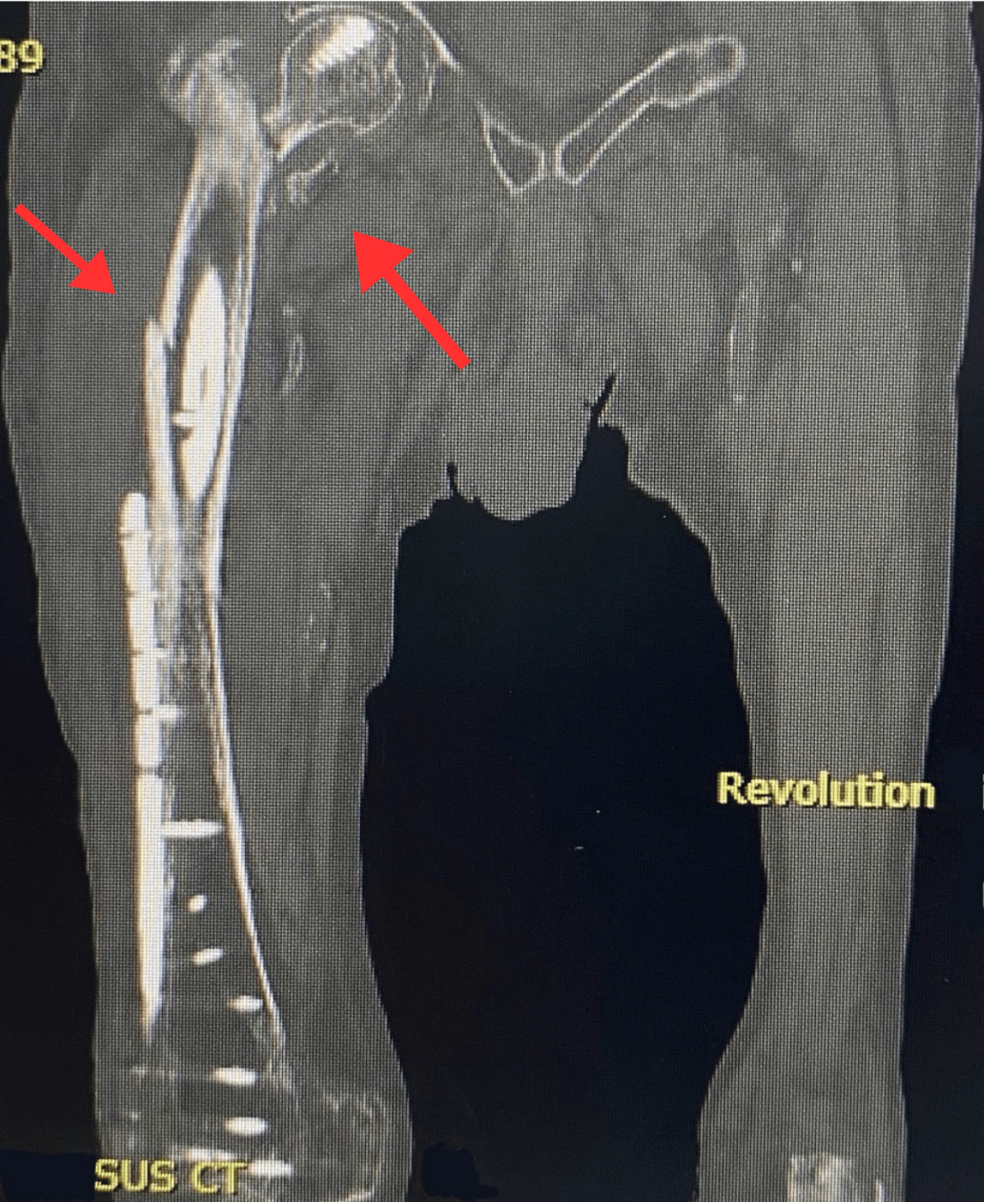
CT scan of the right femur, 17 June 2025, showing a proximal per-implant fracture near the TFNA hardware and evidence of varus malunion with shortening.

Subsequently, a CT scan (Figure 5) performed the same day revealed two critical findings: (1) A per-implant fracture involving the proximal third of the right femur, in proximity to the TFNA hardware, with surrounding hematoma; (2) a healed fracture callus in a varus position with shortening, suggesting malunion contributing to mechanical instability - a finding that was not apparent on the initial X-ray. The presence of a malunited femoral neck fracture combined with the new per-implant fracture led the surgical team to conclude that conversion to hip hemiarthroplasty was necessary rather than attempting further fixation, given the poor bone quality and complex mechanical situation.

**Figure 6:**
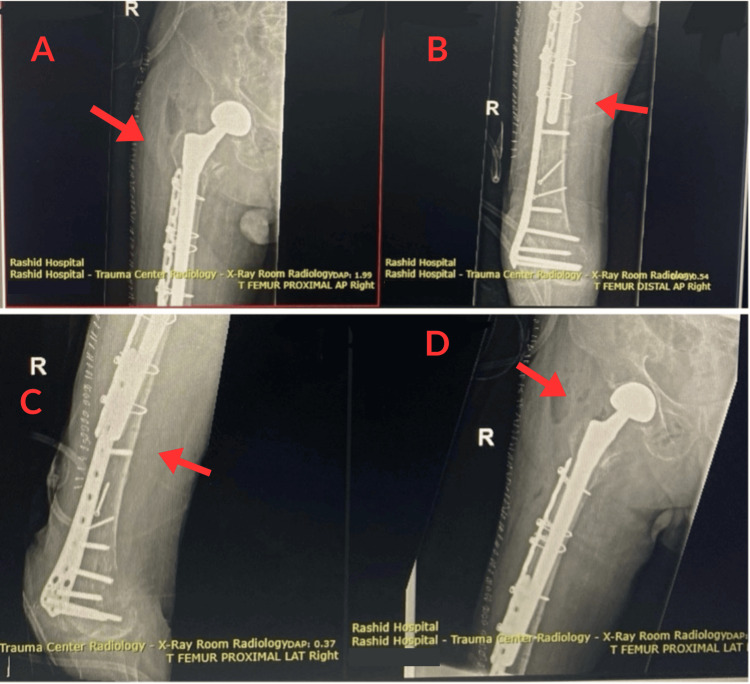
Postoperative right hip X-rays (AP view), 27 June 2025, showing hardware placement and satisfactory alignment after hemiarthroplasty. (A) AP view of the proximal right femur showing the uncemented Wagner SL revision stem and Biolox Delta ceramic head. (B) AP view of the distal right femur demonstrating the lateral locking compression plate extending distally with multiple locking screws. (C) Lateral view of the right femur showing the length and alignment of the locking plate along the femoral shaft. (D) Lateral view focusing on the proximal femur and hip prosthesis, demonstrating proper positioning of the femoral stem and absence of hardware complications.

On 24 June 2025, the patient underwent hardware removal, including extraction of the TFNA nail, lag screw, and proximal screws from the prior distal femoral plate. Using a lateral surgical approach, open reduction and internal fixation of the new fracture were performed with a locking compression plate and multiple cerclage cables. Conversion to hemiarthroplasty was undertaken using an 18/265 mm Zimmer Biomet Wagner SL uncemented modular femoral stem (Zimmer Biomet, Warsaw, IN) and a 28 mm Biolox Delta ceramic femoral head (CeramTec, Plochingen, Germany), selected due to poor proximal bone quality and the need for distal fixation. The stem was inserted uncemented to achieve diaphyseal fixation and minimize cement-related risks given the patient’s comorbidities. The acetabulum was found to be intact and did not require resurfacing. Intraoperatively, care was taken to avoid creating stress risers between the plate and stem, and cerclage cables were used to aid fracture reduction and implant stability. The procedure was completed without intraoperative complications, with an estimated blood loss of 250 mL. Follow-up imaging on 27 June 2025 showed satisfactory alignment of the prosthesis and the plate fixation, with no evidence of complications, as shown in Figure 6A-6D.

At the most recent postoperative assessments in early July 2025, the patient remained medically stable but demonstrated significant functional limitations. Blood pressure readings were persistently low (as low as 92/45 mmHg), and he required moderate to maximal assistance for bed mobility, transfers, and standing. Range of motion of the right hip was limited to 30-50° flexion and 15° abduction, with notable weakness of the hip musculature (1+ to 2/5). Despite participating in physiotherapy and achieving some improvement, the patient could ambulate only short distances with a walker and two-person assistance before experiencing fatigue. Importantly, his mobility was significantly limited not only by residual lower limb weakness but also by a right distal radius fracture requiring immobilization in a back slab, which severely restricted his ability to grip the walker and maintain upper-limb support during gait. This upper limb impairment became a major factor contributing to his delayed mobilization and increased fall risk. Consequently, wheelchair use was advised for longer distances. Pain was reported as mild (Numeric Rating Scale 2-3/10) at the right hip. Rehabilitation efforts continue, focusing on improving muscle power, balance, and gait training.

The patient was discharged on paracetamol and intermittent opioids for pain, along with enoxaparin 40 mg daily for six weeks as deep vein thrombosis prophylaxis, and continues under physiotherapy to improve strength, balance, and gait function. The patient was advised to follow up with endocrinology for osteoporosis management. Initiation of calcium, vitamin D, or antiresorptive therapy was under consideration at the time of discharge, but specific medications were not documented in the available records. At the latest follow-up on 29 July 2025, he resides at home, requires moderate assistance for daily activities, ambulates short distances with a walker, and uses a wheelchair for longer distances, with mild pain managed on intermittent analgesics.

## Discussion

This case highlights several important challenges in managing proximal femoral fractures in elderly patients with significant comorbidities and pre-existing implants. Our patient initially underwent successful internal fixation with TFNA for an intertrochanteric fracture. However, the subsequent development of a per-implant fracture and previously undiagnosed malunion of the femoral neck illustrates how complications may evolve even after apparent initial healing.

Notably, the malunion was occult on plain radiographs and only became evident on CT imaging, consistent with prior reports emphasizing the superior sensitivity of cross-sectional imaging in detecting subtle mechanical failures or malalignment after hip fracture fixation [14,15].

Per-implant fractures in osteoporotic bone, particularly in the context of prior hardware, present significant technical challenges. Further fixation attempts often carry a high risk of failure, and literature suggests that conversion arthroplasty offers a reliable salvage option when mechanical instability or compromised bone quality preclude stable fixation [16,17]. In our patient, conversion to hemiarthroplasty was deemed the most appropriate approach given the malunion, new fracture, and poor bone stock.

In this case, a modular revision stem was selected due to compromised proximal femoral bone quality and the need for secure distal fixation. The Zimmer Biomet Wagner SL stem allows diaphyseal anchorage, bypassing areas of malunion and poor metaphyseal support, which is essential in cases of per-implant fractures or bone loss [18]. The choice of a 28 mm Biolox Delta ceramic femoral head was made to reduce wear and the risk of osteolysis while balancing hip stability and minimizing dislocation risk, consistent with evidence supporting ceramic bearings in elderly patients undergoing hemiarthroplasty [19].

Additionally, the presence of overlapping hardware from previous distal femoral fixation further complicated surgical planning. Sequential femoral surgeries require careful intraoperative management to address implant removal, preservation of bone stock, and restoration of limb alignment.

Despite these complexities, our patient achieved early mobilization and was ambulatory with assistance at follow-up, aligning with evidence that arthroplasty can yield favourable outcomes in similar high-risk scenarios [20]. Nevertheless, his case exemplifies the multifactorial nature of recovery in elderly patients, where medical comorbidities, muscle wasting, and soft-tissue injury all contribute to ongoing functional limitations.

This case underscores the importance of advanced imaging and individualized surgical planning in elderly patients presenting with per-implant fractures and prior hardware.

## Conclusions

This case highlights the complex challenges of managing proximal femoral fractures in elderly patients with osteoporotic bone and prior orthopedic implants. Malunion can remain occult on plain radiographs but may be identified on CT imaging, underscoring the value of advanced imaging in persistent or recurrent symptoms. Per-implant fractures, particularly in poor bone quality, often necessitate conversion to arthroplasty rather than further internal fixation. Sequential femoral surgeries involving overlapping hardware demand meticulous surgical planning and intraoperative adaptability. Optimal outcomes in such high-risk patients require a multidisciplinary approach addressing both orthopedic and medical comorbidities. Awareness of these factors can guide clinicians in making timely and individualized decisions to improve patient function and quality of life.
